# Studium Soziale Arbeit 2035: Eine Einführung in den Schwerpunkt

**DOI:** 10.1007/s12054-022-00493-0

**Published:** 2022-05-30

**Authors:** Katharina Mangold, Wolfgang Schröer

**Affiliations:** Hildesheim, Deutschland

**Keywords:** Zukunft, (duales) Studium, Covid-19, Studi, Co II, Krisen

## Abstract

Einleitung in den Schwerpunkt „Studium 2035“, die die Hauptaspekte der Beiträge kurz skizziert und zudem drei studentische Zukunftsszenarien in Form fiktiver Tagebucheinträge präsentiert.

Mit dem Schwerpunkt „Studium Soziale Arbeit 2035“ wagen wir einen Blick in die Zukunft, stellen Visionen, Szenarien und Befürchtungen nebeneinander und setzen uns mit Veränderungen auseinander. Dabei geht der Blick nicht weit in die Zukunft, sondern gerade mal 13 Jahre – also ein überschaubarer Zeitraum und doch …
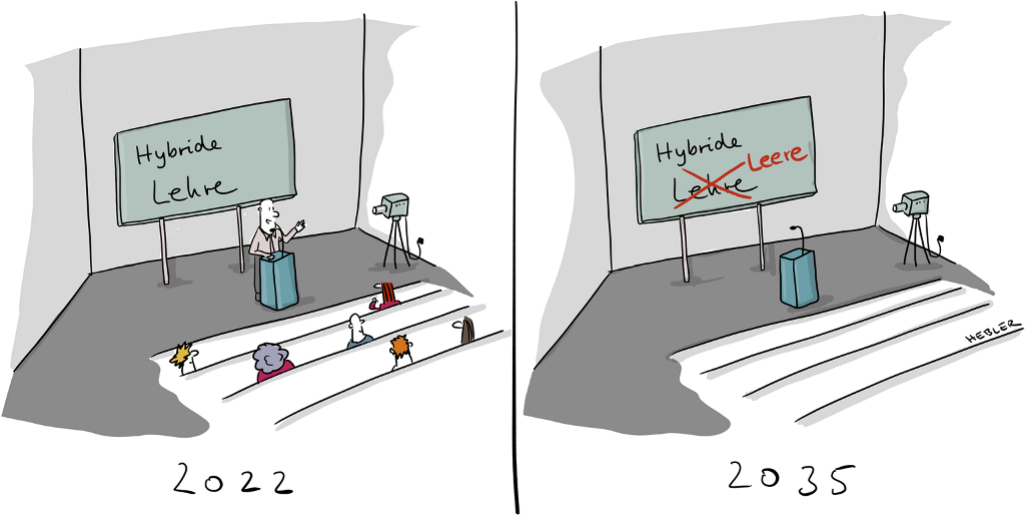


Wer denkt, dass Hochschullehre eingefahren und unveränderbar ist, wurde in den letzten zwei Jahren der Corona-Pandemie eines Besseren belehrt – von heute auf morgen wurde von Präsenz- auf Onlinelehre umgestellt. Sozialarbeiter_innen und Sozialpädagog_innen wurden so vor allem in Online-Formaten ausgebildet. Hat sich dabei grundlegend etwas verändert oder wurden nur – mehr oder weniger gelungen – die bisherigen Lehrangebote in ein Onlineformat gepresst? Was haben wir aus der „Zwangs“-Digitalisierung der Hochschullehre im Rahmen der Corona-Pandemie gelernt und was lässt sich davon für eine Weiterentwicklung des Sozialpädagogikstudiums ableiten? Verdeckt die neue Auseinandersetzung mit Lehrformaten, die wichtige Auseinandersetzung mit aktuellen politischen Herausforderungen sowie theoretischen und professionellen Kernfragen der Sozialen Arbeit?

*Anna Lips und Dorothee Kochskämper* werfen auf Grundlage einer bundesweiten Befragung von Studierenden in den „Corona-Semestern“ (Studie Studi.Co II) einen Blick in die Zukunft. Wie wird das Studium Sozialpädagogik/Sozialarbeit im Jahr 2035 aussehen? Mit welchen Inhalten werden sich angehende Sozialpädagog_innen beschäftigen? Welche didaktischen Methoden werden vorherrschend sein? An welchen Orten lernen Studierende – sitzen sie vor dem PC und interagieren in Kacheln, findet Lernen in Räumen der Hochschulen statt oder auch abseits davon? *Sophie Domann und Birgit Oelker* beschäftigen sich in ihrem Beitrag mit diesen Fragen und zeigen was sich in Bezug auf digitale Lehre in den letzten Jahren entwickelt hat.

Daran anschließend gibt *Kiaras Gharabaghi* in einem Essay einen konkreten Einblick in seine Online-Vorlesung, in der er den bewegten Raum bewusst inszeniert und mit Inhalt verbindet. *Angelika Iser* setzt in ihrem Beitrag die zwischenmenschliche Interaktion in den Fokus und stellt ein Peer-Mentoring-Programm vor, das – digital oder präsent – die individuelle Begleitung von Studierenden durch Studierende im Blick hat und hierbei die Möglichkeit der beraterischen Kompetenzen der Studierenden stärkt.

Schon heute haben wir eine Ausdifferenzierung von Studiengängen, die zwar einen Bezug zur Sozialen Arbeit aufweisen, in ihren jeweiligen Titeln jedoch weit differenzierter sind. Die Berufspraxis erlebt dies häufig als undurchschaubaren Dschungel und weiß mit so manchem Studienabschluss nichts mehr anzufangen bzw. ihn nicht einzuordnen. Das Modell der Staatlichen Anerkennung als Sozialpädagog_in/Sozialarbeiter_in scheint hier ein Versuch zu sein, diese Vielfalt in ein Gütesiegel zu zertifizieren und darüber abzubilden, ob der Studiengang grundlegend sozialpädagogisch war. Was wird aber mit dem politisch immer wieder auch umstrittenen Modell der Staatlichen Anerkennung sein? Hat sie sich als Gütesiegel überholt? Entstehen andere Formate der begleiteten Berufseinmündung nach dem Studium? *Inka Janssen* diskutiert das Model der Staatlichen Anerkennung. In diesem Zusammenhang stellt sich auch stets die Frage vom gemeinsamen Lernort Hochschule und Fachpraxis bzw. der Wechselseitigkeit und Bedingtheit dieser beiden Lernorte. Hiermit beschäftigt sich *Gunther Graßhoff* am Beispiel von dualen Studiengängen.

Ein Blick in die Zukunft macht uns möglicherweise auch deutlich, dass wir rückblickend blind waren für bestimmte Themen und Entwicklungen. So zeigt unser Interview mit *Mechthild Wolff*, wie wichtig Schutzkonzepte an Hochschulen sind und dass insbesondere Soziale Arbeit als Disziplin, die stets Teil der Entwicklung von Schutzkonzepten und Reflexion von Machtprozessen in der sozialpädagogischen Praxis ist, auch selbst Schutzkonzepte in der eigenen Organisation entwickeln und vorlegen muss. Doch ist dies in den aktuellen organisationspolitischen Modellen von Hochschulen überhaupt möglich?

Der Schwerpunkt fokussiert auf organisationale und mediale Fragen rund um das Studium. Hierbei werden jedoch auch soziale und professionelle Themen und Inhalte sichtbar, mit welchen sich Soziale Arbeit in den nächsten Jahren und Jahrzehnten beschäftigen wird, weil sie auf gesellschaftliche Veränderungen reagieren muss. So kommen wir nicht umhin, uns intensiv mit Fragen des Klimawandels zu beschäftigen und den sich darin reproduzierenden Formen globaler soziale Ungleichheiten. Zudem muss sich Soziale Arbeit hier fragen lassen, wie junge Menschen in einer Welt von Krisen (klimatisch, militärisch, weltpolitisch …) gestärkt werden können. Konkrete Fragen von Schutz und Macht, Partizipation, Friedensarbeit und Beteiligung als Inhalte des Studiums, der Profession und als Struktur des Studiums selbst müssen im Zentrum der Überlegungen stehen.

Nach der intensiven Beschäftigung mit diesem Schwerpunkt haben wir uns als dessen Koordinator_innen erlaubt, abschließend drei studentische Szenarien in Form von Tagebucheinträgen für den 5. Juni 2035 zu entwerfen:

- *„Dienstag, den 5. Juni 2035: Heute habe ich mich auf meine Abschlussprüfung vorbereitet. Während ich mir ein academic video zur Kinder- und Jugendhilfe angeschaut habe, habe ich es mir draußen in meiner Hängematte bequem gemacht. Danach war ich mit zwei Kommilitoninnen joggen und wir haben darüber diskutiert. Das war interessant, weil für mich ganz andere Themen relevant waren als für meine Kommilitoninnen. Im Austausch habe ich dann nochmals mehr gelernt. Danach habe ich über diese Unterschiede mit meinem ‚personal advisor‘ gechattet und wir haben viel darüber gesprochen, dass ich stets, mit dem was mich aktuell prägt und beschäftigt, auf das neue Wissen treffen. Ich habe verstanden, dass ich – gerade in diesen beschleunigten und krisenhaften Zeiten – im Studium auch herausgefordert bin, mich mit mir und meinem Erfahrungswissen auseinander zu setzen. Das ist oft ziemlich mühsam und vor allem meine Idee die digitalen Module auf in unserem ‚social-work-study-circle‘ in zwei Jahren zu beenden, geht nicht mehr ganz auf. Da digital auch einiges synchron und zeitlich flexibel studiert werden kann, dachte ich zuerst, dass ich Nebenjob, Reisen und Studium gut unter einen Hut bekomme und das Ganze recht schnell durchziehen kann. Klar ist es möglich, das Wissen jederzeit von jedem Ort der Welt anzuhören und anzueignen und von der Möglichkeit mache ich auch Gebrauch, wenn ich beispielsweise nächste Woche meinen Großeltern pflege, weil meine Eltern – die das sonst machen – im Urlaub sind.“*
- *„Dienstag, den 5. Juni 2035: Gerade habe ich mir im Zug ein Video von unserer Hochschule reingetan, das gerade da lief. Da wurde soon Professor verabschiedet. Der jammerte herum, dass sie früher in so etwas wie Vorlesungen noch Studierenden Zusammenhänge im Hörsaal persönlich erklärt hätten und sie vor allem viel miteinander vor Ort diskutiert hätten. Dieses Gejammere habe ich lange mehr gehört. Ich studiere zwar schon zehn Semester neben meinen Jobs und meinem Engagement in einer Initiative gegen Zensur und Rassismus. Ja, jetzt gehen sie wohl die letzten Dinosaurier, die irgendwie verloren wirkten und immer wieder der sogenannten Covid-19-Krise von vor fünfzehn Jahren alles in die Schuhe schieben. Auf dem Abschiedsvideo wird auch gesagt: ‚Viel zu schnell sei es damals gegangen, viel zu wenig sei diskutiert worden, ob jetzt alles digital werden sollte. Es sei doch nur ein erneuter neoliberaler Versuch gewesen, die Hochschule weiter zu ökonomisieren.‘ Neo … was? Ja, ich kann mir schon vorstellen, dass es nicht leicht ist, die Hochschule von heute zu begreifen. Die reden auch immer von digitaler Hochschule, ich weiß gar nicht, was eine nicht-digitale Hochschule sein soll. Kann ich mir nicht vorstellen, wie soll das denn gehen? Für jeden Sch … Energie verschwenden und zur Hochschule fahren, um jemanden zu treffen und dem Papier geben, so viel Bäume können wir doch gar nicht fällen, steht doch längst auf dem Umweltschutzindex … doch ich wünsche dem alten Professor alles Gute. Eigentlich war der ganz OK und hat sich auch für uns eingesetzt – doch auch etwas jammerig …“*
- *„Dienstag, den 5. Juni 2035: Heute Nachmittag hatte ich Seminar am Campus und wir haben intensiv über den Zusammenhang von Klima und Flucht und die damit verbundenen Herausforderungen für Soziale Arbeit diskutiert. Gut, dass das Studium der Sozialen Arbeit als einziger Studiengang unserer Hochschule nicht vollkommen digitalisiert wurde. Nach dem Seminar hab ich mich im Rahmen der Peer-to-Peer-Beratung mit einer Studierenden getroffen, die ich nun als Peer-Beraterin schon länger kenne und sie hat mir berichtet, wie sie sich in Prüfungen ungerecht behandelt und bedrängt fühlt. Ich konnte die Studierende darin bestärken die Ansprechpersonen, die im Rahmen des hochschulweiten Schutzkonzeptes existieren, anzusprechen und werden sie bei dem Onlinetermin begleiten. Dann hatte ich etwas Stress, um noch rechtzeitig zur FIAN-Regionalgruppe zu kommen, wo wir uns für globale Nahrungsgerechtigkeit einsetzen. Jetzt muss ich noch eine Aufgabe meines Portfolios ins Learnweb laden, weil die Abgabe heute um 23:59 Uhr schließt. Ich habe in der vergangenen vorlesungsfreien Zeit ein Praktikum im Frauenhaus gemacht und dieses dann in Interaktion mit einem Online-Programm, das auf mein Praxisfeld programmiert war, reflektiert. Hierbei habe ich mich nochmals mit einem Text zur Intersektionalität auseinandergesetzt, das ist ein Ansatz aus den 2000er Jahren, ganz schön alt, aber immer noch aktuell, finde ich, den ich immer wieder neu verstehen muss … Ein Freund, der bereits im Jugendamt arbeitet, hat mir gesagt, dass das ganz normal sei und dass man nie ‚fertig‘ ist und er nach wie jedem Tag ein 10-minütiges Online-Learning-Nugget anhört, das ihn immer wieder neue Ideen und Gedanken für seinen Arbeitsalltag bringt. Seine Kolleg_innen schaffen das nicht immer“*.

